# Transcriptome analysis of novel B16 melanoma metastatic variants generated by serial intracarotid artery injection

**DOI:** 10.1186/s40478-025-01924-1

**Published:** 2025-01-16

**Authors:** Jenny C. Kienzler, Erick M. Contreras, Janet Treger, Linda M. Liau, Geoffrey C. Owens, Robert M. Prins

**Affiliations:** 1 Department of Neurosurgery, University of California, Los Angeles, CA USA; 2 Department of Molecular and Medical Pharmacology, University of California, Los Angeles, CA USA; 3 Jonsson Comprehensive Cancer Center, University of California, Los Angeles, CA USA; 4 Parker Institute for Cancer Immunotherapy Center at UCLA, Los Angeles, CA USA; 5 Present Address: Institute of Experimental Immunology, University of Zurich (UZH), Winterthurerstrasse 190, 8057 Zurich, Switzerland; 6 Department of Neurosurgery, University Hospital of Lausanne, Lausanne, Switzerland; 7 Present Address: University of California, Riverside, CA USA

**Keywords:** Melanoma, B16-F0, Brain metastasis, Leptomeningeal disease, Epithelial to mesenchymal transition, Lipid rafts

## Abstract

The incidence of brain metastases (BrM) in patients with metastatic melanoma is reported to be 30–50% and constitutes the third most frequent BrM after breast and renal cancers. Treatment strategies including surgical resection, stereotactic radiation, and immunotherapy have improved clinical response rates and overall survival, but the changes that occur in circulating melanoma cells to promote invasion of the brain are not fully understood. To investigate brain tropism, we generated new variants of the B16 mouse melanoma model by serially passaging B16 cells through the brain of immune competent syngeneic C57BL/6 mice. Cells were injected into the right carotid artery and recovered from the brain after the mice had reached the study endpoint due to tumor burden, then expanded in vitro and reinjected. We compared the transcriptomes of 4th generation B16 cell populations from separate lineages with the founder B16-F0 cells. Gene set enrichment analysis (GSEA) of differentially expressed protein coding genes revealed that cells isolated from the brain as well as from the lung and meninges expressed higher levels of genes associated with an epithelial to mesenchymal transition (EMT), upregulation of the KRAS signaling pathway, and a metastasis aggressiveness gene signature associated with poor survival in melanoma patients. Principal component analysis of differentially expressed genes showed that 4th generation melanoma cells isolated from the brain, lung and meninges from one lineage were distinct from those of the other three lineages. Among the differentially expressed genes, transcript levels of several genes, including *Itgb2*, *Rftn2*, and *Kcnn4*, were significantly higher in all cell populations that comprised this lineage compared with all cell populations from the other three lineages. In conclusion, we have derived an aggressive, highly brain metastatic B16 variant associated with leptomeningeal disease by serially passaging cells in vivo.

**Supplementary Information:**

The online version contains supplementary material available at 10.1186/s40478-025-01924-1.

## Introduction

Melanoma patients have a high risk of developing metastases in different organs including the brain. It is estimated that the incidence of brain metastases (BrM) in patients with metastatic melanoma is 30–50% [8, 14]. Neurological symptoms from melanoma BrM may be aggravated by local hemorrhage, which can occur in up to 40% of cases [58]. Treatment modalities for melanoma BrM include surgical resection and stereotactic radiosurgery if central nervous system (CNS) involvement is limited, and whole brain radiation if it is more extensive [2, 35]. Among systemic treatment modalities single or combination immunotherapies have been trialed [23, 25]. Leptomeningeal disease (LMD) is associated with ~ 10% of late-stage melanoma [5], however based on autopsy studies this may be an underestimate [50]. Melanoma patients diagnosed with LMD have a very poor prognosis despite new treatment modalities [13].

There is a long history of developing metastatic variants of B16 melanoma cells [1, 18, 19]. These metastatic variants arose from either tail vein injections or spontaneously from cells placed subcutaneously in the flank of the mouse. Intracarotid injection has emerged as an effective model for brain metastasis research. This approach selectively targets cancer cells to the brain, preventing unwanted tumor formation in facial regions and ensuring tumor establishment specifically in the brain. The method better recapitulates the metastatic process by requiring cancer cells to naturally traverse the blood–brain barrier, providing enhanced physiological relevance compared to direct intracranial injection [37]. In the present study we targeted the brain by injecting B16-F0 cells into the right carotid artery of syngeneic C57BL/6 mice, then reinjecting cells recovered from brain metastases for four successive cycles. We compared the transcriptomes of fourth generation B16 cells grown from brain, lung and meninges and found significant changes in gene expression compared with the B16-F0 cells, including increases in a core group of transcripts of genes associated with the extracellular matrix, and KRAS signaling. We identified several new candidate genes that may be associated with aggressive brain metastases.

## Materials and methods

### Surgical procedure

**Fig. 1:**
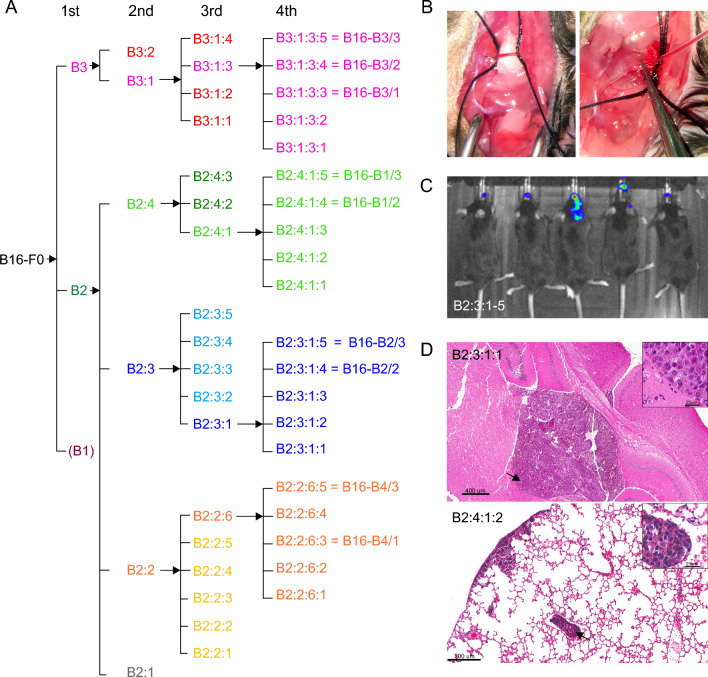
Derivation of B16 melanoma variants by serial intracarotid artery injection. **B** Images from the surgical procedure in which B16 cells are delivered through a polyethylene tube inserted into the carotid artery, **C** chemiluminescence images of the five 3rd generation B16-B2 animals (B2:3:1, B2:3:2, B2:3:3, B2:3:4, B2:3:5) showing tumor growth in the head, **D** H&E sections of brain and lung from two 4th generation animals showing metastatic B16 tumor cells in both organs (arrows point to the locations of the higher power insets), **A** schematic outline of the derivation of the B16 melanoma variants which entailed recovering B16 cells from brain tissue and growing them for four successive generations. The final designations of the 4th generation cell lines that were used for RNAseq are shown

Institutional approval was obtained for the survival surgeries. B16-F0 melanoma cells were injected into the carotid artery of C57BL/6 female mice using a surgical technique that has been described previously [61]. In brief, mice were anesthetized by intraperitoneal injection of ketamine (100 mg/kg) and xylazine (10 mg/kg). After proper placement of the mouse on a glass plate and securing the extremities with tape, neck hair was shaved, and the skin disinfected by applying povidone-iodine and 70% alcohol. The mouse was then placed under the microscope. After skin incision with a surgical scalpel and placement of a spreader, blunt dissection of the muscle with forceps followed to expose the right carotid artery. A 6–0 silk suture was placed proximal and distal to the injection site before a small saline moistened cotton ball was placed below the carotid artery to elevate the vessel at the intended site of injection and to reduce the blood flow. Then a sharp pair of micro-scissors was used to perform a small opening in the carotid artery, followed by the insertion of a polyethylene tube with a sharpened tip (PE10, inner diameter 0.28 mm, outer diameter 0.61 mm). After correct placement of the tube was confirmed by blood regurgitation into the tube, 100 ul of tumor cells resuspended in PBS were injected (Fig. 1A). Successful delivery of the tumor cells could be observed by color change in the nearby arteries for a few seconds. The catheter was then withdrawn, and the proximal and distal placed sutures were used for ligation of the artery. Finally, the situs was closed and the skin sutured with Prolene 4–0 suture. Tumor growth was monitored by bioluminescence imaging. In vivo imaging was performed under isoflurane anesthesia after IP injection of luciferin (100 μl of 5 mg/ml stock solution). Chemiluminescence images were captured using an IVIS Lumina II imaging system (Perkin Elmer, Waltham, MA).

### Cell culture methods

The B16-F0 primary melanoma tumor cell line [18] (American Type Culture Collection, Manassas, VA), and in vivo derivatives were grown in DMEM with 10% heat-inactivated fetal bovine serum (FBS) in 5% C0_2_. To monitor tumor growth in vivo B16-F0 cells were transduced with a dicistronic third generation HIV-based lentivirus encoding strawberry fluorescent protein and firefly luciferase. To recover cells from melanoma BrM, whole brains from injected euthanized mice were dissected and dissociated by enzymatic digestion with Miltenyi® tumor dissociation kit for 45 min (Miltenyi Biotec, Gaithersberg, MD). After adding DMEM media, cells were filtered through a 100 μm filter, spun down and washed with PBS. The resulting cell pellet was resuspended in DMEM containing 10% FBS and penicillin–streptomycin. Dissociated cells were grown in T75 flasks until they reached confluency (6–23 days) prior to reinjecting them into animals. The following numbers of cells were used for the serial injections: 1st generation 1,000,000 cells/100ul, 2nd, 3rd and 4th generation 500,000 cells/100ul.

### RNA extraction and histology

B16-F0 melanoma and 4th generation B16 cells isolated and expanded from brain, lung and meninges were flash frozen, and RNA was extracted using the Zymo® quick RNA kit (Zymo Research, Irvine, CA). Cells were isolated from four different lineages (Fig. 1B). To avoid batch effect, all samples were processed for bulk RNA sequencing (RNA-Seq) at the same time. Whole brains and lungs from 4th generation injected animals were fixed, paraffin embedded and sectioned for H&E staining.

### Library preparation and DNA sequencing

The integrity of RNA preps was verified using the Agilent 4200 TapeStation System (Agilent Technologies, Santa Clara, CA). Libraries for RNA-Seq were constructed with KAPA Stranded mRNA-Seq Kit to generate strand-specific RNA-seq libraries (Roche Diagnostics Corp, Indianapolis, IN). The workflow consists of poly(A) RNA selection, RNA fragmentation and double-stranded cDNA generation using a mixture of random and oligo(dT) priming, followed by end repair to generate blunt ends, adaptor ligation, strand selection, and PCR amplification to produce the final libraries. Amplified libraries were quantified by Qubit dsDNA HS (High Sensitivity) Assay Kit (ThermoFisher, Waltham, MA), and quality-checked by the Agilent 4200 TapeStation System. Different index adaptors were used for multiplexing samples in one sequencing lane. Sequencing was performed with HiSeq3000 sequencer to produce 50 base-pair single-end reads (1 × 50 bp) (Illumina Inc., San Diego, CA).

### Bioinformatics methods

RNAseq data were processed using Partek Flow® software (Partek Inc., St. Louis, MO). Reads were mapped to the latest UCSC transcript set using STAR—2.7.2a [16] and mm10 (GRCm38.97), and gene counts were normalized by Trimmed Mean of the M-values. Pairwise gene set enrichment analysis (GSEA) [54] was performed between brain-derived, lung-derived and meninges-derived B16 cells and B16-F0 cells respectively. 2023 releases of the molecular signatures database were used. Gene lists comprised the genes that were expressed in every sample of either brain-derived, lung-derived or meninges-derived B16 cells, and B16-F0 cells. Conversion to human orthologs used a web function, https://www.syngoportal.org/convert [29]. Differentially expression of individual genes between cell samples was determined using edgeR [45]; cutoffs of ≥ 2 log_2_ fold difference, FDR < 0.05, and *p* < 0.05 were applied (Additional File 1). Metascape [63] was used to assign differentially expressed genes to established gene pathways. NetworkAnalyst 3.0 was utilized to construct a STRING protein–protein interaction network with a high confidence score (0.9) [55, 62]. A network of the most highly correlated differentially expressed genes was constructed using Graphia [21]. Principal components analysis of differentially expressed genes was performed with the R package FactoMinerR [34]. The Cancer Genome Atlas (TCGA) data were obtained through the UALCAN web portal (https://ualcan.path.uab.edu) [10, 11]. Networks were formatted in Cytoscape [48] and exported as scalable vector graphic files to CorelDraw (Corel Corporation, Ottawa, ON). Heatmaps were generated using Morpheus (https://software.broadinstitute.org/morpheus/) and exported to CorelDraw as portable document format files.

## Results

### *Serial *in vivo* transfer of B16-F0*

The starting point for this study was the parental B16-F0 line [17]. A schema outlining the derivation of the different B16 cell lines by serial passage in vivo is shown in Fig. 1A. Cells were injected into the carotid artery (Fig. 1B), and as exemplified in Fig. 1C, establishment of tumors at each step was verified by in vivo imaging. When the mice reached the endpoint due to tumor burden (14–23 days after injection), they were euthanized and metastases from the brain were dissociated, expanded until confluent, and then vitally frozen. For the second generation, the two most brain-homing cell lines by observation were chosen for intracarotid injections (B2 and B3; Fig. 1A). Four brain-derived B16 lines were selected from this second generation for the next round of injections (Fig. 1A). During the surgical procedure to generate the 3rd generation of B16 derivatives the injection was either made into the common carotid artery, or the external carotid artery was ligated prior to the injection, and cells were selectively injected into the internal carotid artery. For a final in vivo transfer, four 3rd generation B16 derivatives were injected into the common carotid artery (B2:2:4, B2:3:3, B2:4:2, and B3:1:3; Fig. 1A). The B16-B3 lines showed by observation more of a brain preference in comparison to the other cell lines, starting at the second generation. Third generation B16-B3 cells were very brain distinct and showed less tumor growth in the skull base and the lungs, irrespective of whether the external carotid had been ligated. This observation also pertained to the 4th generation B16-B3 cells with fewer lung metastases compared with the other B16 derivatives, although this was not quantified. In agreement with previous work [46], we only observed B16 cells in meninges and brain ventricles not in the parenchyma (Fig. 1D).

### Transcriptome analysis

**Fig. 2:**
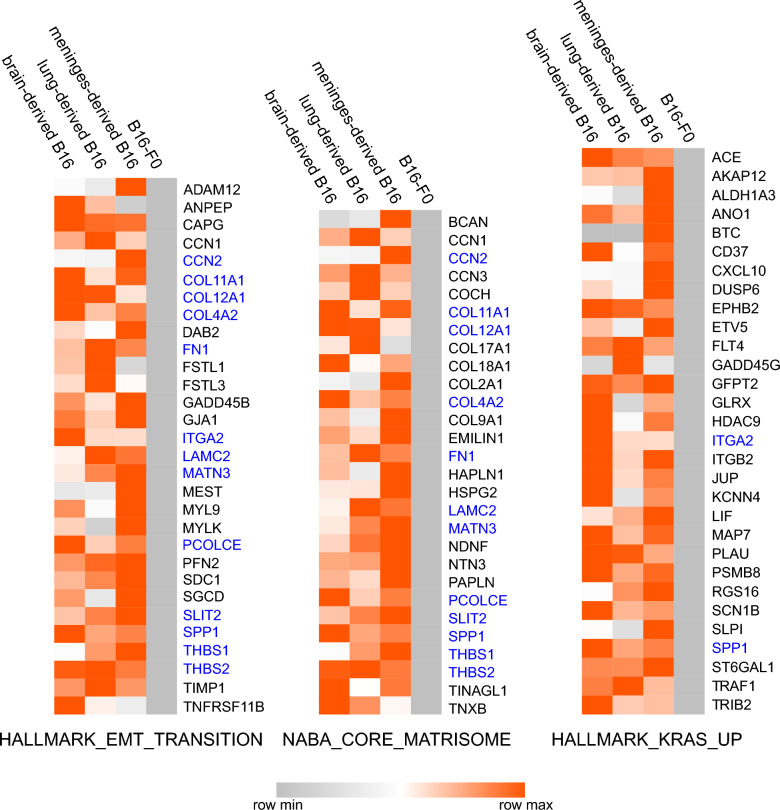
Increased transcript levels of genes associated with EMT, Matrisome, and the KRAS signaling pathway. Median normalized transcript counts from melanoma cells isolated from brain, lung and meninges compared with three independently grown populations of B16-F0. Genes were selected that were found in each pairwise GSEA analysis between B16-F0 and B16-derived brain, lung and meninges cells with a rank metric score > 1. Overlapping genes between the three molecular signatures are shown in blue. Medians are re-scaled from 0 to 1

Transcript data were obtained from 4th generation melanoma cells grown from brain, meninges and lung, and three independently grown populations of B16-F0 cells. For GSEA, only the protein coding genes were used, and only those that were expressed in every sample (Additional file 1). Comparisons were made with both the murine and human signature gene sets, and B16 cells derived from all three tissues were significantly enriched in genes associated with an epithelial to mesenchymal cell transition (EMT), a core signature of cytoskeletal proteins associated with aggressive melanoma metastases in human [59], and upregulation of the KRAS signaling pathway (Fig. 2). The corresponding enrichment plots are shown in Additional file 2, and heat maps of the log_2_ normalized counts of the genes in all three comparisons in all samples with a rank score > 1 are shown in Additional file 3.

**Fig. 3:**
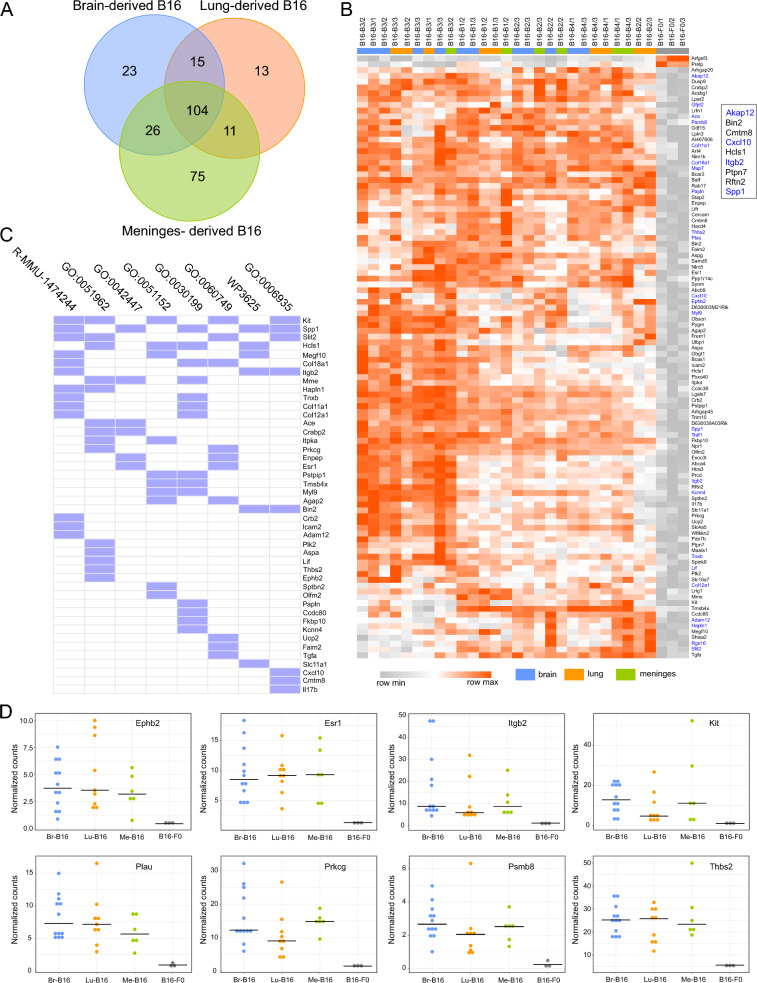
Identification of a common set of genes in the B16 variants. **A** Venn diagram showing that there are 104 genes whose expression is highly significantly different from B16-F0 in all tissue derived B16 cells (> log_2_ two-fold difference, p < 0.05, FDR < 0.05), **B** heatmap showing the expression of the 104 genes in every sample of 4th generation B16 cells. (log_2_ normalized counts re-scaled from 0 to 1). Hierarchical clustering of rows and columns is based on the 1-Pearson correlation coefficient metric and average linkage method. Boxed genes are differentially expressed in human metastatic melanoma; genes in blue are associated with upregulation of the KRAS signaling pathway, **C** association of the 104 genes with Reactome, Gene Ontology and Wikipathways databases (log_10_P < − 3.5): R-MMU-1474244, Extracellular matrix organization; GO:0051962, positive regulation of nervous system development; GO:0042447, hormone catabolic process; GO:0051152, positive regulation of smooth muscle cell differentiation; GO:0030199, collagen fibril organization; GO:0060749, mammary gland alveolus development; WP3625, Tyrobp causal network in microglia; GO:0006935, chemotaxis, **D** dot plots of normalized transcript counts of potential hub genes among the 104 genes, based on a String PPI network (0.9 confidence limit)

We made pairwise comparisons of individual genes between 4th generation tissue-derived melanoma cells and the parent F0 cells and identified a common set of 104 genes whose expression differed by > twofold between the 4th generation melanoma cells and F0 cells (Fig. 3A and B; Additional file 1). Approximately 40% of these genes were associated with established functional pathways in particular a set of mouse genes associated with extracellular matrix organization (Fig. 3C). We also compared the list of 104 genes to the TCGA database and identified nine genes that are expressed at a significantly higher level in human metastatic melanomas (n = 368) compared with primary tumors (n = 104) (> twofold difference in median expression; transcript per million > 1). These genes are listed in the inset in Fig. 3B, and boxplots taken from UALCAN web portal are shown in Additional file 4. *AKAP12*, *CXCL10, ITGB2*, and *SPP1* are all associated with upregulation of the KRAS pathway (Fig. 2).

To identify potential hub genes among the 104 differentially expressed genes we constructed a protein: protein interaction (PPI) network based on the STRING database of functional gene associations. The genes with the most connections to other genes in the B16 cell transcriptome would be candidate hub genes (Additional file 5). The expression levels of the most highly connected genes in the PPI network are plotted in Fig. 3D and includes *ITGB2*. These genes may be involved in controlling the metastatic behavior of the B16 variants.

**Fig. 4:**
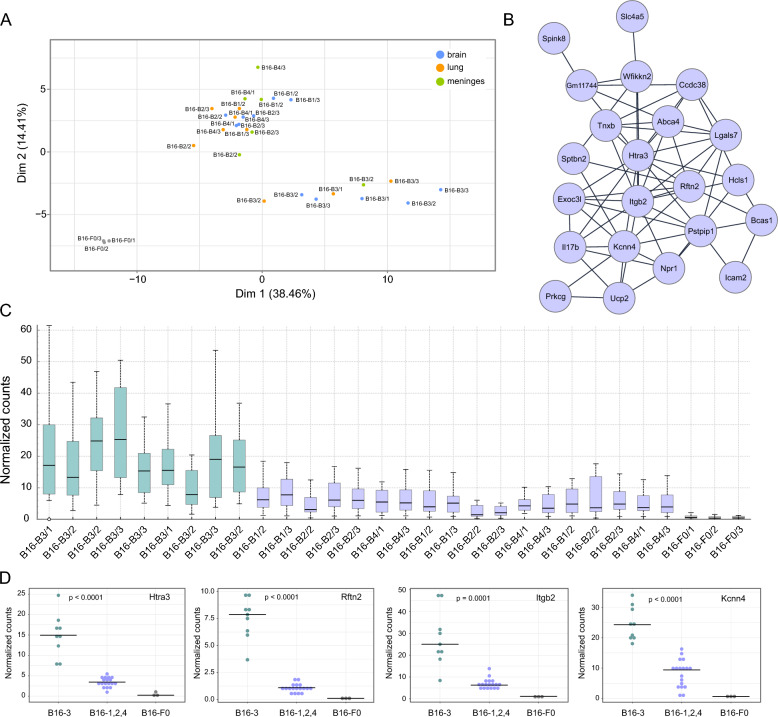
B16 variants partition into two distinct groups. **A** A PCA plot calculated from the normalized counts of transcripts of the 104 core genes shows that the 4th generation B16-B3 variant differs from the B16-B1, B16-B2, and B16-B4 variants, **B** network constructed from the most highly correlated core genes shown in Fig. 3B (Pearson correlation coefficient > 0.95, cluster size > 5), **C** interquartile box plot calculated from the normalized counts of all the genes in the network shown in (B), **D** dot plots of the normalized transcripts counts of those genes in the network shown in (B) that are the most connected

Principal component analysis using the normalized gene counts of the 104 genes clearly separated the tissue-derived cells into two groups, reflecting the source of the cells, B16-B3 versus B16-B1, -B2, -B4 (Fig. 4A). We also used linear correlation and Louvain clustering to construct a network of the co-expressed genes among the 104 genes (Pearson correlation coefficient of > 0.95; > 5 genes per cluster) (Fig. 4B). This network was expressed at a higher level in B16-B3 melanoma cells, suggesting that they may account in part for the observation that B16-B3 cells appeared to show an increased propensity to target the brain compared to B16-B1, B2, and B4 cells (Fig. 4C). As shown in Fig. 4D, expression of the most highly connected genes was significantly different between the B16-B3, and B16-B1, -B2, -B4 cells. Higher levels of *RFTN2* and *ITGB2* transcripts are also found in metastatic human melanomas compared with primary skin tumors (Additional file 4).

## Discussion

Following the in vivo-in vitro selection approach developed by Isiah Fidler [17, 19] we have generated four new polyclonal B16 variants. To preferentially target the brain, we injected B16-F0 cells into the common carotid artery, and only selected cells that had seeded the brain. In agreement with Schackert and Fidler [46] we found that B16 cells grew extensively in the meninges and brain ventricles but not in the brain parenchyma per se. Intracarotid artery injection of human melanoma cell lines in immune deficient mice also leads to metastases in the meninges and ventricles [31, 47, 49]. The involvement of the meninges in melanoma BrM is well established [13, 24, 50, 53], and the prognosis for melanoma patients with LMD is very poor. High resolution MRI has revealed that small intracranial melanoma metastases are often found in close association with the pial surface suggesting that some parenchyma metastases may originate in the leptomeninges [33]. Although there are very few reported cases of melanoma BrM with ventricular involvement, the prognosis is again very poor [3, 6, 9, 27] We therefore believe that these new B16 variants in particular B16-B3 will be valuable models for LMD associated with melanoma BrM.

Analysis of RNA transcripts from 4th generation B16 cells isolated from brain, lung and meninges revealed significant changes in gene expression compared with the parental B16-F0 line that were consonant with a more aggressive metastatic phenotype. Our analysis was carried out on cells that were expanded in vitro, indicating that the changes in gene expression we observed were maintained after multiple cell divisions ex vivo. The phenotypic stability of polyclonal B16 metastatic variants in vitro was previously described by Isiah Fidler’s group [41]. We conjecture that in vivo serial transfer of B16 cells results in epigenetic modification of selected genes resulting in heritable changes in gene expression and the development of stable variants [12]. On the other hand, a recent comparison of established B16-F0 and B16-F10 cells by single cell RNA-seq revealed the presence of putatively pro-metastatic subpopulations in the B16-F0 cells [28]. This would suggest that existing variants possibly fixed by mutation in the parental B16-F0 population are selected for by serial transfer in vivo. In future work, a single cell ATAC-seq experiment with our B16 variants should resolve these questions.

The expression of *SPP1* (osteopontin), a regulator of epithelial-mesenchymal transition (EMT) [30] and a characteristic of melanomas with high metastatic potential [40], was approximately ten-fold higher in our B16 variants compared with the parental B16-F0. It has been reported that knockdown of *SSP1* in melanoma cells results in significantly reduced tumor growth [15], and in a B16 mouse melanoma model, SPP1 blockade suppressed melanoma metastasis [26]. In addition to *SPP1*, GSEA showed that other genes linked to KRAS activation were expressed at higher levels in the brain-derived B16 variants, including the scaffolding protein gene *AKAP12* (A-kinase anchoring protein 12), whose increased expression in human metastatic melanomas correlates with reduced survival [20]. *KRAS* has been reported to be a potential driver gene for melanoma BrM [42]. In addition, *BIN2, CMTM8*, *CXCL10*, *HCLS1*, *ITGB2*, *PTPN2*, and *RFTN2* were expressed at significantly higher levels in our B16 brain-derived variants compared with the parent B16-F0 and are also upregulated in human metastatic melanomas compared with primary cutaneous tumors. (Additional file 4).

Following injection into the common carotid artery, B16 cells would be expected to move from the internal branch into blood vessels in the dura and choroid plexuses. Although the endothelial cells of the blood vessels in the dura and choroid plexus are fenestrated, there are tight junctions between the epithelial cells that form the arachnoid barrier under the dura, and on the ventricular side of the choroid plexus [22, 57]. Therefore, B16 cells have to traverse these barriers in order to enter the subarachnoid space and ventricles. It appears that the B16 cells may use the same paracellular and/or transcellular means as leukocytes to enter the CSF as indicated by the marked increase in expression of *ITGB2* in the brain-derived B16 variants compared with the parent B16-F0. Notably, in B16-B3 cells isolated from the brain *ITGB2* transcripts were up to 45-fold higher. Integrin β2 dimerizes with integrin αL to form leukocyte-function-associated antigen 1 (LFA-1), which binds intercellular adhesion molecules (ICAMs) on endothelial cells as part of the process of diapedesis [44]. ICAMs are also expressed on the apical side of choroid plexus epithelial cells [52], indicating a potential interaction between the B16 variants in the ventricles and the epithelial cells lining the ventricles. Complement C3 has been shown to be involved in the growth of tumor cells in CSF [7]. Because C3 transcripts were not detected in every sample it was excluded from our differentially gene expression analysis. It may however be significant that integrin dimers comprising ITGB2 bind iC2b, a downstream cleavage product of C3 [60].

We found that B16-B3 cells were distinguished from B16-B1, 2 and 4 cells by a group of genes whose high expression levels were significantly correlated. Three of the most connected genes in this network were *ITGB2*, *KCNN4* and *RFTN2*. *KCNN4* (KCa3.1) encodes a calcium-activated potassium channel that has been was shown to promote invasion and metastasis in hepatocellular carcinoma [36]. In clear renal cell carcinoma increased expression of *KCNN4* has been linked to shorter progression-free and overall survival, and a high metastatic potential [43]. The use of selective KCa3.1 channel inhibitors to treat cancers is an area of active research [4, 38, 56].

*RFTN2* encodes Raftlin family member 2 a major component of lipid rafts, which are microdomains in membranes enriched in cholesterol, sphingolipids, gangliosides that form platforms for signaling via integrins, ion channels and receptors [32, 39]. Thus, high co-expression of *RFTN2*, *ITGB2*, and *KCNN4* and other genes in the network may reflect an increase in the formation of the lipid rafts and consequent enhanced signaling. Isolation and proteomic analysis of lipid rafts from the B16 variants and parent F0 cells would address this supposition.

### Limitations

Further work is required to test the functional significance in melanoma brain metastases of the genes that we have identified in this study. In addition, injection of these new variants into more distal and peripheral sites will be required to evaluate preferentially homing to the brain. Other groups have shown that selected B16 melanoma variants that metastasize to the same organ do not necessarily show an organ specificity [51]. Since we found that intracarotid artery injection of 4th generation brain-derived B16 cells still leads to seeding of the lungs, we doubt that the B16 variants reported in this study would only invade the brain following subcutaneous placement or tail vein injection. But we believe that these new cell lines reflect well the phenotype of a melanoma that seeds the brain in metastatic leptomeningeal disease.

## Conclusion

We generated novel aggressively metastatic B16 melanoma cell lines by serial in vivo-in vitro transfer and have identified genes whose elevated expression compared with the original B16-F0 cell line may account for an enhanced metastatic phenotype. These genes are associated with migration, invasiveness, and proliferation, and reflect an epithelial to mesenchymal transformation and engagement of KRAS signaling. The involvement of lipid rafts in brain metastases is strongly suggested by our gene analysis. Our data identified multiple targets that can be validated in human melanoma brain metastases samples. These new B16 cell lines, in particular B16-B3, will be useful for further preclinical studies and to test new approaches to treating brain metastases associated with leptomeningeal disease.

## Supplementary Information

Additional file 1

Additional file 2

Additional file 3

Additional file 4

Additional file 5

## Data Availability

The datasets used and analyzed during the current study available from the corresponding author on reasonable request.

## Abbreviations

BrM: Brain metastases
CNS: Central nervous system
EMT: Epithelial to mesenchymal transition
GSEA: Gene set enrichment analysis
ICAMs: Intercellular adhesion molecules
LMD: Leptomeningeal disease
TCGA: The cancer genome atlas
