# Supplementary material for: Transcriptome analysis of novel B16 melanoma metastatic variants generated by serial intracarotid artery injection
Source: Acta Neuropathol Commun. 2025 Jan 16;13:10. doi: 10.1186/s40478-025-01924-1 (PMC11737150; doi:10.1186/s40478-025-01924-1)

Additional file 2: Gene set enrichment analysis

Brain-derived B16 v B16-F0

NES = 1.89, nominal p value = 0.000, FDR q value = 6.631E-4

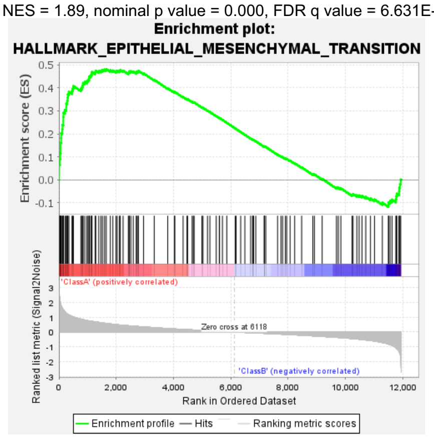

NES = 2.06, nominal p value = 0.000, FDR q value =0.000

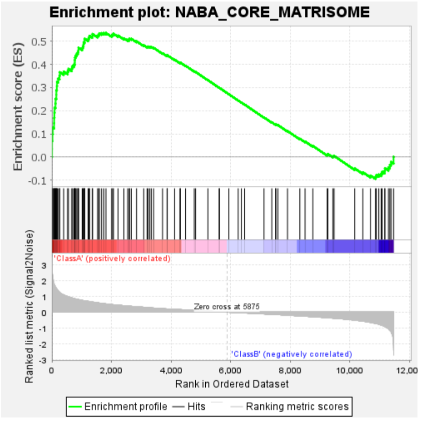

NES = 2.17, nominal p value = 0.000, FDR q value =0.000

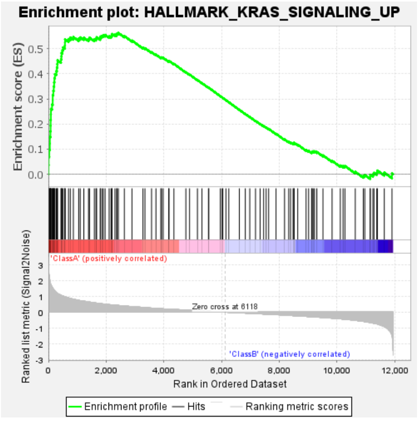

Lung-derived B16 v B16-F0

NES = 2.06, nominal p value = 0.000, FDR q value =0.000

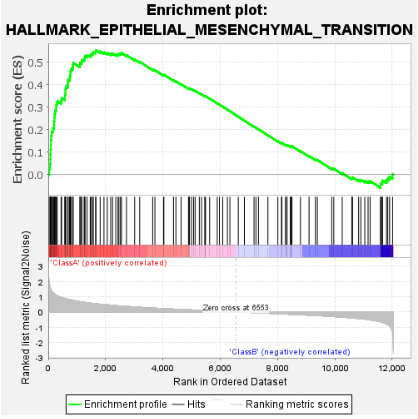

NES = 2.17, nominal p value = 0.000, FDR q value =0.000

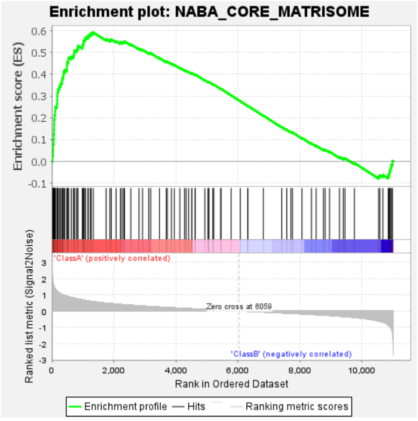

NES = 2.31, nominal p value = 0.000, FDR q value =0.000

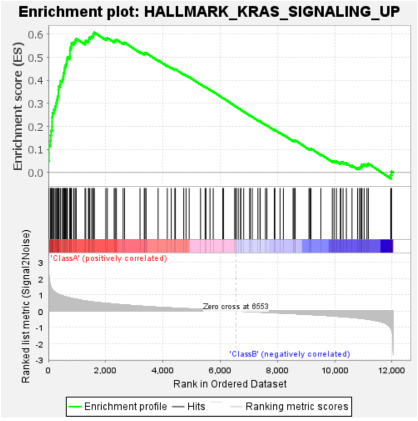

Meninges-derived B16 v B16-F0

NES = 1.95, nominal p value = 0.000, FDR q value =6.78E-4

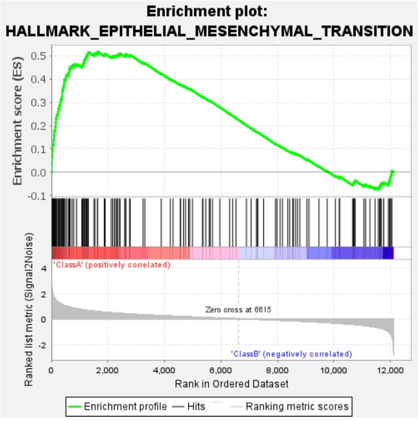

NES = 2.16, nominal p value = 0.000, FDR q value =0.000

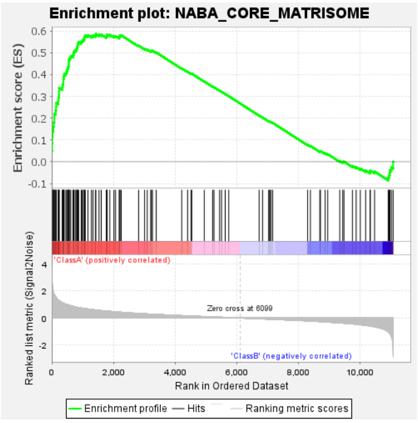

NES = 2.11, nominal p value = 0.000, FDR q value =0.000

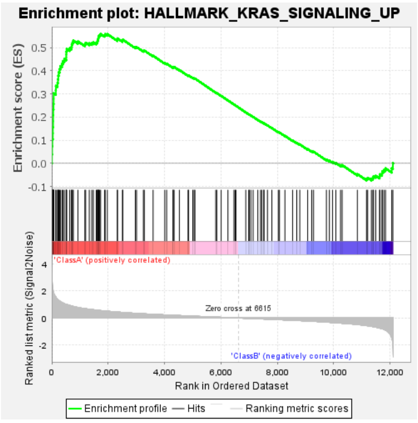

Supplement: Supplementary file 2 — Additional file 2 [file 40478_2025_1924_MOESM2_ESM.pdf]
