# Supplementary material for: Transcriptome analysis of novel B16 melanoma metastatic variants generated by serial intracarotid artery injection
Source: Acta Neuropathol Commun. 2025 Jan 16;13:10. doi: 10.1186/s40478-025-01924-1 (PMC11737150; doi:10.1186/s40478-025-01924-1)
Supplement: Supplementary file 3 — Additional file 3 [file 40478_2025_1924_MOESM3_ESM.pdf]

Additional file 3: Heat maps

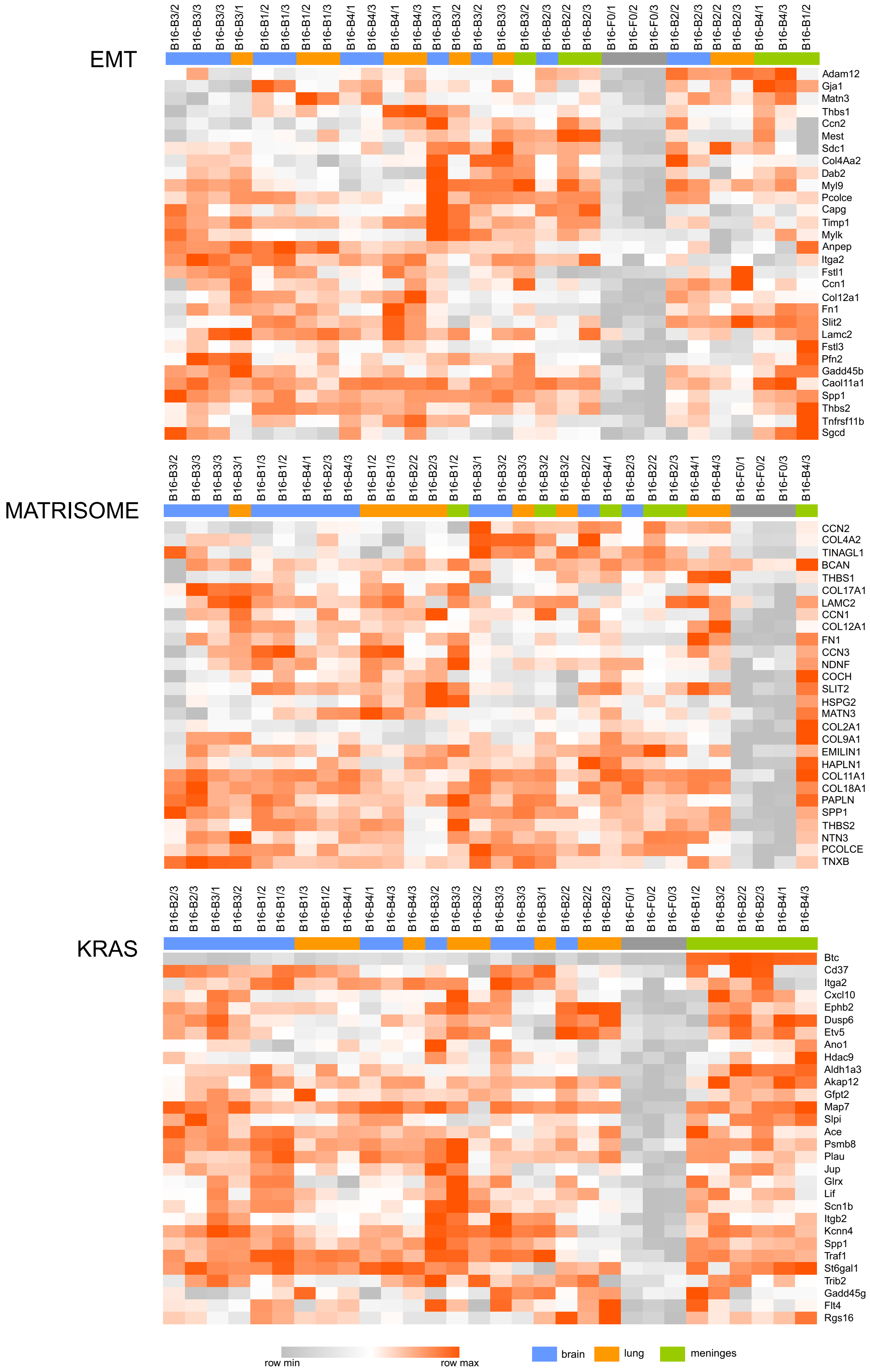

Heat maps of log2 normalized counts of top core genes with a rank metric score >1 from the GSEA. Hierarchical clustering of columns and rows using a 1- Pearson correlation coefficients as the distance metric and clustering using the average linkage method.
