# Supplementary figures and images for: Transcriptome analysis of novel B16 melanoma metastatic variants generated by serial intracarotid artery injection
Source: Acta Neuropathol Commun. 2025 Jan 16;13:10. doi: 10.1186/s40478-025-01924-1 (PMC11737150; doi:10.1186/s40478-025-01924-1)

## Additional file 4: Box plots of TCGA data

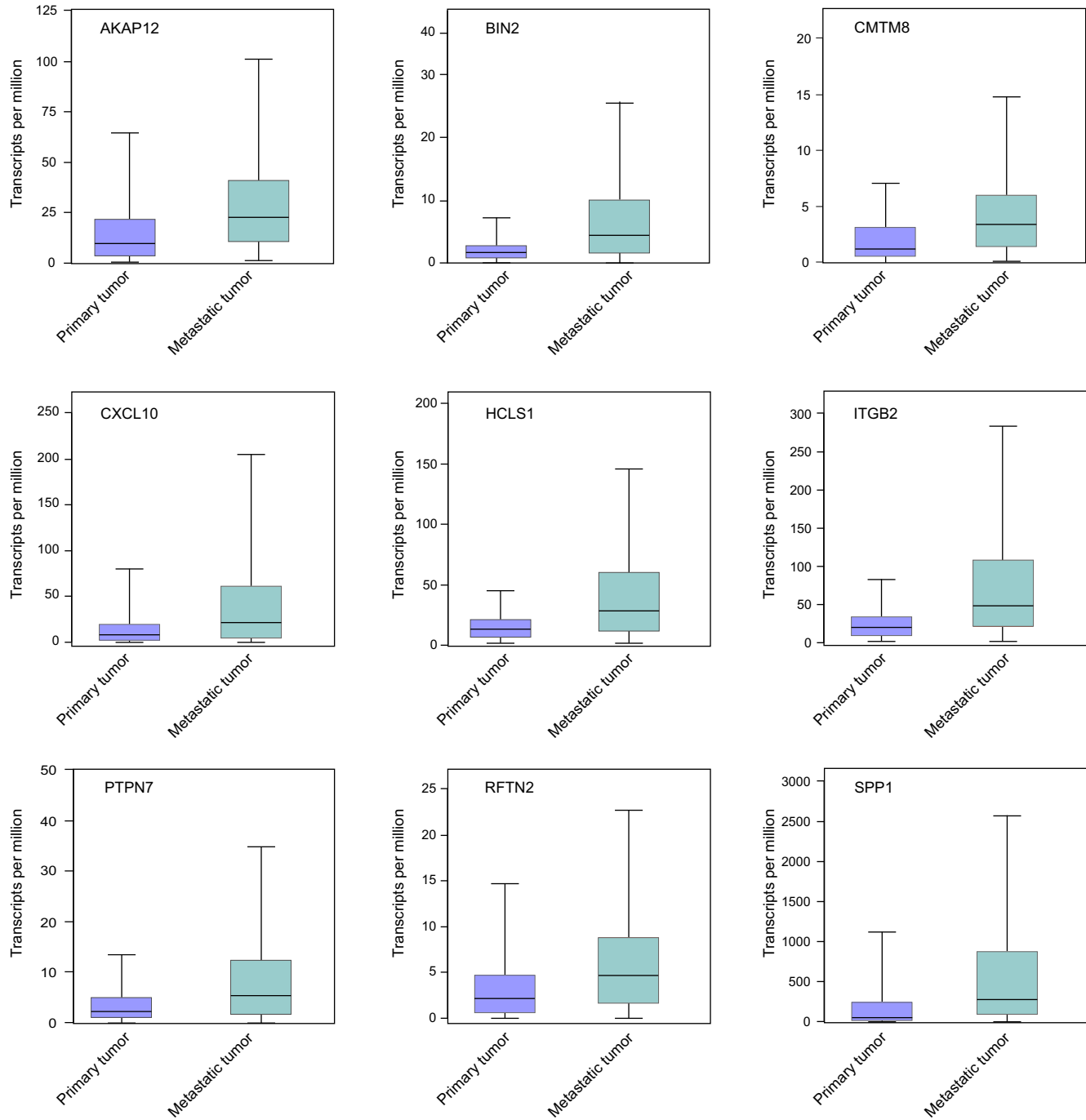

Supplement: Supplementary file 4 — Additional file 4 [file 40478_2025_1924_MOESM4_ESM.pdf]
