# Supplementary material for: Transcriptome analysis of novel B16 melanoma metastatic variants generated by serial intracarotid artery injection
Source: Acta Neuropathol Commun. 2025 Jan 16;13:10. doi: 10.1186/s40478-025-01924-1 (PMC11737150; doi:10.1186/s40478-025-01924-1)
Supplement: Supplementary file 5 — Additional file 5 [file 40478_2025_1924_MOESM5_ESM.pdf]

# Additional file 5: PPI network

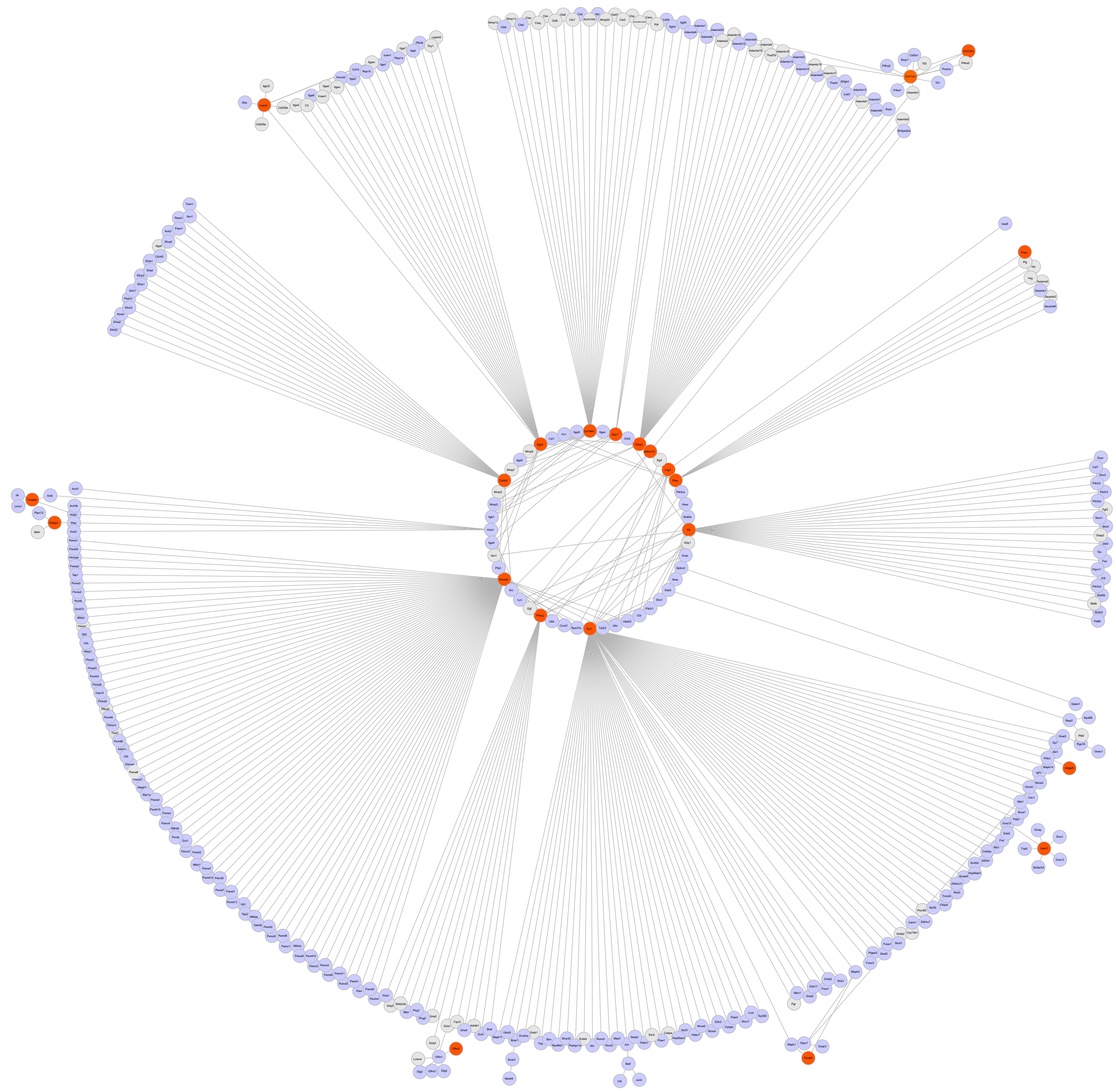

The human orthologs of the 104 differentially expressed genes were input into NetworkAnalyst to generate a network of protein: protein interactions (PPI) based on the STRING database. Each spoke in the network represents a high confidence functional association.

Gene from list of 104 differentially expressed genes (Figure 3)

Gene from list of genes expressed in B16 cells (Additional File 1)

Gene not found in the list of expressed genes (Additional File 1)
